# Supplementary material for: Mediation of a Mutualistic Conflict for Pollination via Fig Phenology and Odor Recognition between Ficus and Fig Wasp
Source: Plants (Basel). 2022 Oct 3;11(19):2603. doi: 10.3390/plants11192603 (PMC9572538; doi:10.3390/plants11192603)
Supplement: Supplementary file 1 [file plants-11-02603-s001.zip › plants-1925496-supplementary.pdf]

Supplementary Materials

Mediation of a mutualistic conflict for pollination via fig phenology and odor recognition between *Ficus* and fig wasp

Wen-Hsuan Chen[1,2,†], Anthony Bain[3,4,†], Sheng-Yang Wang[1, 5], Yi-Chiao Ho[1,6] and Hsy-Yu Tzeng[1,*]

[1] Department of Forestry, National Chung Hsing University, Taichung 40227, Taiwan
[2] Chiayi Forest District Office, Forestry Bureau, Council of Agriculture, Executive Yuan.
[3] Department of Biological Sciences, National Sun Yat-Sen University, Kaohsiung, Taiwan
[4] International PhD Program for Science, National Sun Yat-sen University, Kaohsiung 80424, Taiwan
[5] Academy of Circular Economy, National Chung Hsing University, Taichung 40227, Taiwan
[6] Hsinchu Forest District Office, Forestry Bureau, Council of Agriculture, Executive Yuan
† These authors contributed equally to this work.
* Correspondence: erecta@dragon.nchu.edu.tw; Tel.: +886-4-22840345 ext. 142.

Table S1. The relative amount and occurrence of volatile compounds in receptive female and male *Ficus septica* figs.

	(Female n=9)		Male (n=14)	
Compounds	Occurrence	Relative amount (%)	Occurrence	Relative amount (%)
**Benzenoids**				
Benzaldehyde	1	05.69±16.10	2	00.80±01.99
**Monoterpene**				
Alloocimene	3	01.66±02.57	4	00.65±01.29
Cis-ocimene	2	00.45±00.84	1	00.17±00.63
Cyclosativen	0		1	00.36±01.31
Eucalyptol	4	15.51±21.08	9	22.09±22.84
Linalool	5	09.83±20.21	12	18.44±23.89
Neoalloocimene	2	01.56±03.27	4	01.41±02.29
Sabinene	6	10.90±09.70	9	05.92±06.61
Terpinolene	2	00.63±01.25	1	00.04±00.15
Trans-α-ocimene	3	10.67±15.20	2	04.01±11.23
Trans-β-ocimene	1	00.25±00.69	3	02.18±04.75
α-Pinene	4	04.38±08.10	3	01.46±03.33
α-Terpinen	1	00.18±00.50	0	
β-pinene	1	00.31±00.86	4	07.63±25.64
γ-Terpinene	2	00.61±01.17	1	00.12±00.42
1,3,8-P-Menthatriene	2	00.28±00.60	1	00.06±00.20
**Sesquiterpenes**				
Aromadendrene	0		1	00.11±00.40
Germacrene D	0		2	01.44±04.53
Valencene	0		4	02.67±05.90
α-Caryophyllene	0		1	00.31±01.12
α-Copaene	2	02.54±05.32	6	06.70±18.25
α-Gurjunene	0		4	03.78±08.42
β-Caryophyllene	0		1	01.02±03.69
β-Elemene	1	00.60±01.69	5	00.35±00.58
δ-Elemene	0		2	00.25±00.69
**Unknown**				
A	0		1	00.17±00.62
B	0		2	03.67±11.52
C	0		1	00.73±02.64
D	1	09.00±25.45	1	00.87±03.12
E	1	05.42±15.32	0	
F	1	01.91±05.40	1	00.59±02.11
G	1	00.30±00.83	1	00.95±03.42
H	1	00.27±00.76	2	00.28±00.69
I	1	04.34±12.27	4	01.41±02.31
J	1	10.12±28.63	1	04.22±15.22
K	1	02.55±07.22	4	04.90±08.03
L	1	00.05±00.13	1	00.21±00.75

*The detected compounds are divided into three groups based on general biosynthetic origin (Knudsen et al., 2006). Relative amounts: mean ± SE of the proportion of each compound in the total bouquet (%) and for each compound, the number of trees in which the compound was detected (occurrence).

Table S2. PCA axis scaled to unit length, based on the sample occurrences and relative amount of the different volatile bouquets of fig at receptive stage.

	Relative amount (%)		
compounds	Axis 1	Axis 2	Axis 3
1,3,8-P- Menthatriene	-0.0020	0.0022	-0.0005
A	-0.0038	-0.0005	0.0012
Alloocimene	-0.0170	0.0129	-0.0032
Aromadendrene	0.0038	0.0023	0.0050
B	0.0739	-0.0807	-0.0276
Benzaldehyde	0.0113	0.0582	-0.0389
C	-0.0161	-0.0019	0.0051
Cis-ocimene	-0.0047	0.0037	-0.0009
Cyclosativen	0.0054	0.0033	-0.0053
D	0.0272	0.1242	-0.0863
E	0.0295	0.0564	-0.0417
Eucalyptol	-0.7854	-0.1531	0.2330
F	0.0506	-0.0735	-0.0126
G	0.0008	0.0009	-0.0032
Germacrene D	-0.0248	-0.0016	0.0060
H	-0.0157	-0.0029	0.0051
I	-0.0231	0.0315	-0.0046
J	0.3975	0.3976	0.6648
K	-0.2172	-0.0448	0.0742
L	-0.0022	0.0019	-0.0008
Linalool	0.3578	-0.8295	0.0261
Neoalloocimene	-0.0106	0.0094	-0.0064
Sabinene	0.0019	0.054	0.0056
Terpinol	-0.0072	0.0025	0.0003
Trans-α-ocimene	-0.1141	0.0866	-0.0209
Trans-β-ocimene	0.0086	-0.0189	-0.0060
Valencene	0.0061	0.0110	-0.0129
α-Caryophyllene	0.0012	0.0000	-0.0011
α-Copaene	0.1261	-0.0230	-0.0785
α-Gurjunene	0.0089	0.0316	-0.0276
α-Pinene	0.0304	0.0516	0.0212
α-Terpinen	-0.0016	0.0006	0.0002
β-Caryophyllene	0.0088	-0.0165	-0.0027
β-Elemene	0.0126	0.0194	0.0271
β-pinene	0.0880	0.2784	-0.6905
γ-Terpinene	-0.0051	0.0041	-0.0008
δ-Elemene	0.0004	0.0025	-0.0018

First and second eigenvectors these can be used as coordinates in a distance-based biplot (Fig 2), where the distances among objects approximate their Euclidean distances.
